# Penicillamine Increases Free Copper and Enhances Oxidative Stress in the Brain of Toxic Milk Mice

**DOI:** 10.1371/journal.pone.0037709

**Published:** 2012-05-21

**Authors:** Ding-Bang Chen, Li Feng, Xiao-Pu Lin, Wei Zhang, Fu-Rong Li, Xiu-Ling Liang, Xun-Hua Li

**Affiliations:** 1 Department of Neurology, First Affiliated Hospital, Sun Yat-sen University, Guangzhou, China; 2 Nanfang Hospital, Southern Medical University, Guangzhou, China; 3 Laboratory Animal Centre, Sun Yat-sen University, Guangzhou, China; Center of Ophtalmology, Germany

## Abstract

Wilson disease (WD) is characterized by the accumulation of copper arising from a mutation in the ATP7B gene. Penicillamine (PA) makes 10–50% of the patients with neurologic symptoms neurologically worse at the early stage of administration. The aim of this study was to determine how the copper metabolism changes and whether the change impairs the brain of toxic milk (tx) mice, an animal model of WD, during the PA administration. The free copper and protein-bound copper concentrations in the serum, cortex and basal ganglia of tx mice with PA administration for 3 days, 10 days and 14 days, respectively, were investigated. The expression of copper transporters, ATP7A and CTR1,was analyzed by real-time quantitative PCR, immunofluorescence and Western blot. Then SOD, MDA and GSH/GSSG were detected to determine whether the oxidative stress changed correspondingly. The results revealed the elevated free copper concentrations in the serum and brain, and declined protein-bound copper concentrations in the brain of tx mice during PA administration. Meanwhile, transiently increased expression of ATP7A and CTR1 was observed generally in the brain parenchyma by immunofluorescence, real-time quantitative PCR and Western blot. Additionally, ATP7A and CTR1 were observed to locate mainly at Golgi apparatus and cellular membrane respectively. Intense staining of ATP7A in the choroid plexus was found in tx mice on the 3rd and 10th day of PA treatment, but rare staining of ATP7A and CTR1 in the blood-brain barrier (BBB). Decreased GSH/GSSG and increased MDA concentrations were also viewed in the cortex and basal ganglia. Our results suggested the elevated free copper concentrations in the brain might lead to the enhanced oxidative stress during PA administration. The increased free copper in the brain might come from the copper mobilized from brain parenchyma cells but not from the serum according to the ATP7A and CTR1 expression analysis.

## Introduction

Wilson disease (WD) is an autosomal recessive disease arising from a mutation in the ATP7B gene, which results in the failure of hepatocytes to excrete copper into bile, leading to the hepatic copper accumulation and cell injury. Eventually copper is released into the bloodstream and deposits in various other organs, notably the brain, kidneys, and cornea. Copper incorporation into the protein ceruloplasmin (Cp) is also impaired by dysfunction of ATP7B, leading to the reduced circulating holoceruloplasmin.

The treatment for WD patients involves reversing the positive copper balance. Chelators like penicillamine or trientine induce negative copper balance by cupriuresis [1]. The pivotal role of penicillamine in the initial treatment for WD patients with neurologic symptoms has been a matter of debate for the risk of neurological worsening for the past three decades. 10%–50% of patients neurologically worsened by PA and 50% of them never recovered to the level before treatment [2]. Trientine shares the propensity of penicillamine to deteriorate neurologic symptoms (about 25%) [3].

Previous study reported that free copper increased in the cerebrospinal fluid (CSF) and serum in WD patients with the neurologic manifestation during the penicillamine therapy [4]. Neurons were found an early and extensive death when treated with copper in vitro, which mirrored the concentration of the CSF of WD patients in previous report [5]. These findings indicated the increased free copper concentration in the brain might associate with the neurologic deterioration. The likely explanation for the drugs worsening the neurological symptoms was that a further elevation of copper level in the brain followed the mobilization of large stores of copper in the liver and/or the brain [4], [6], [7]. But there has been no direct evidence supporting the hypothesis yet.

Copper transport into cells is mediated by copper transporter1 (CTR1), which mediates copper uptake into mammalian cells, through the copper-stimulated endocytosis and degradation of CTR1 [8]. ATP7A and ATP7B are both copper transport proteins which participate in copper efflux out of cells,whereas ATP7A is the dominating copper transporter in neurons [9]. ATP7A moves towards the plasma membrane and removes excess copper in the presence of elevated extracellular copper concentrations [10]. Earlier studies reported the expression of the copper transpoters,ATP7A and CTR1, was higher in brain barriers than that in brain parenchyma by in situ brain perfusion technique with copper, and the copper transported into the brain was mainly achieved through the blood-brain barrier(BBB) [11], [12].

In the serum copper is mainly carried by Cp, while the residue bound to albumin or small molecules. Free copper, which is commonly defined as the copper not covalently bound to Cp in the blood, is loosely bound to albumin and small molecules. Free copper, which is about 5–15% of serum copper in normal people, greatly expanded in WD patients and diffused out of the vascular compartment into extracellular fluids and tissues with toxic effects [13].The free copper concentration is assessed with the calculation of the serum non Cp-bound copper [1]. However, it was suggested copper transported into the brain through the blood-brain barrier (BBB) as free copper ions but not as albumin-bound copper [11]. The estimation of copper concentration by the traditional method didn't eliminate the role of albumin or other proteins which bound unnecessary copper and lessened the toxic effect.

The toxic milk (tx) mouse is a naturally occurring genetic and phenotypic model of WD derived from the wild-type (DL) mouse [14]. The mouse contains a methionine to valine substitution (M1356V) that renders the ATP7B protein dysfunctional and results in copper accumulation, which commences in the 3rd postnatal week, in a distribution resembling that observed in human WD cases. An increase of copper in the liver and the brain has been well characterized in tx mice [15]. In this study we investigated the concentrations of free copper and protein-bound copper in the serum and the brain tissue, separately, by a different method of ultrafiltration during PA treatment [16]. The expression of ATP7A and CTR1 was also detected to estimate the copper transporting activity in the brain. Furthermore we examined the possible neurotoxicity during the treatment. Superoxide dismutase (SOD) and reduced glutathione/oxidized glutathione (GSH/GSSG) ratio were measured as intracellular indicators of antioxidant responses, while the malondialdehyde (MDA) content as a lipid peroxidation index.

## Results

### Copper concentrations

**Figure 1:**
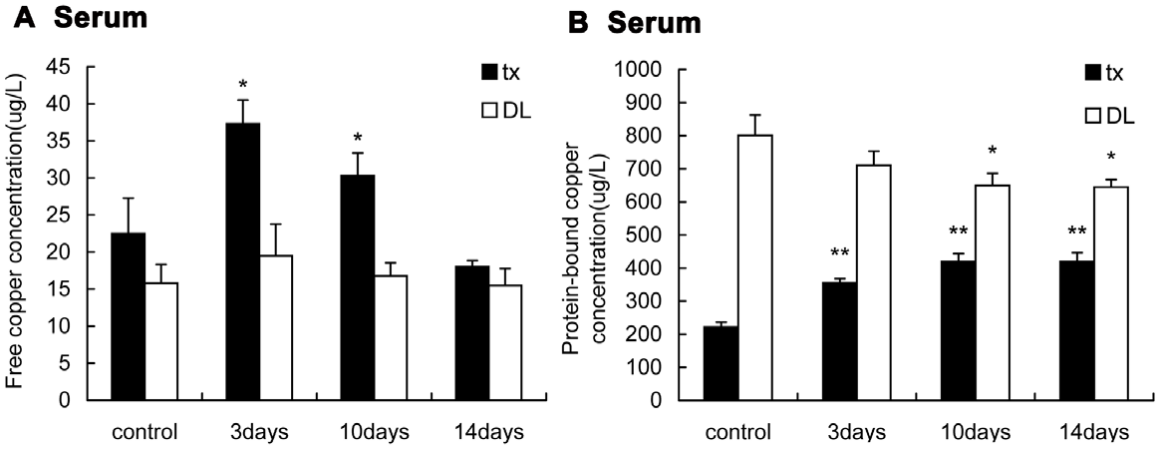
Detection of the free copper and protein-bound copper in the serum of tx and DL mice. Tx and DL mice were subjected to PA intragastric administration for 3 days, 10 days and 14 days, respectively. Mice without PA were controls. (A)The free copper concentrations in the tx mice serum increased on the 3rd day, and decreased on the 10th and the 14th day. The free copper concentrations didn't change significantly in DL mice. (B)The protein-bound copper concentrations in the serum 2-fold increased on the 3rd day compared to the controls and kept the level on the 10th and the 14th day, while slightly decreased along the administration in the serum of DL mice.(* P<0.05;** P<0.01).

The average serum free copper concentration detected by ultrafiltration method was about 8.9% of serum copper concentration, the sum of free copper and protein-bound copper, in control group of tx mice,while in DL mice it was 1.9%. The free copper concentrations in tx mice serum significantly elevated on the 3rd day after persistent PA administration (P<0.05), then declined progressively and below the level of control mice on the 14th day (Fig. 1). Obviously the free copper concentrations in DL mice were lower than tx mice and also lightly increased 3 days after PA administration (P>0.05). The protein-bound copper concentrations in tx mice serum were significantly lower than that in DL mice. In tx mice the mean protein-bound copper concentrations was nearly 200 ug/L and almost 2-fold increased on the 3rd day of PA treatment and kept the level up to the 14th day (P<0.01). In normal mice the copper bound to protein was slightly decreased during the PA treatment.

**Figure 2:**
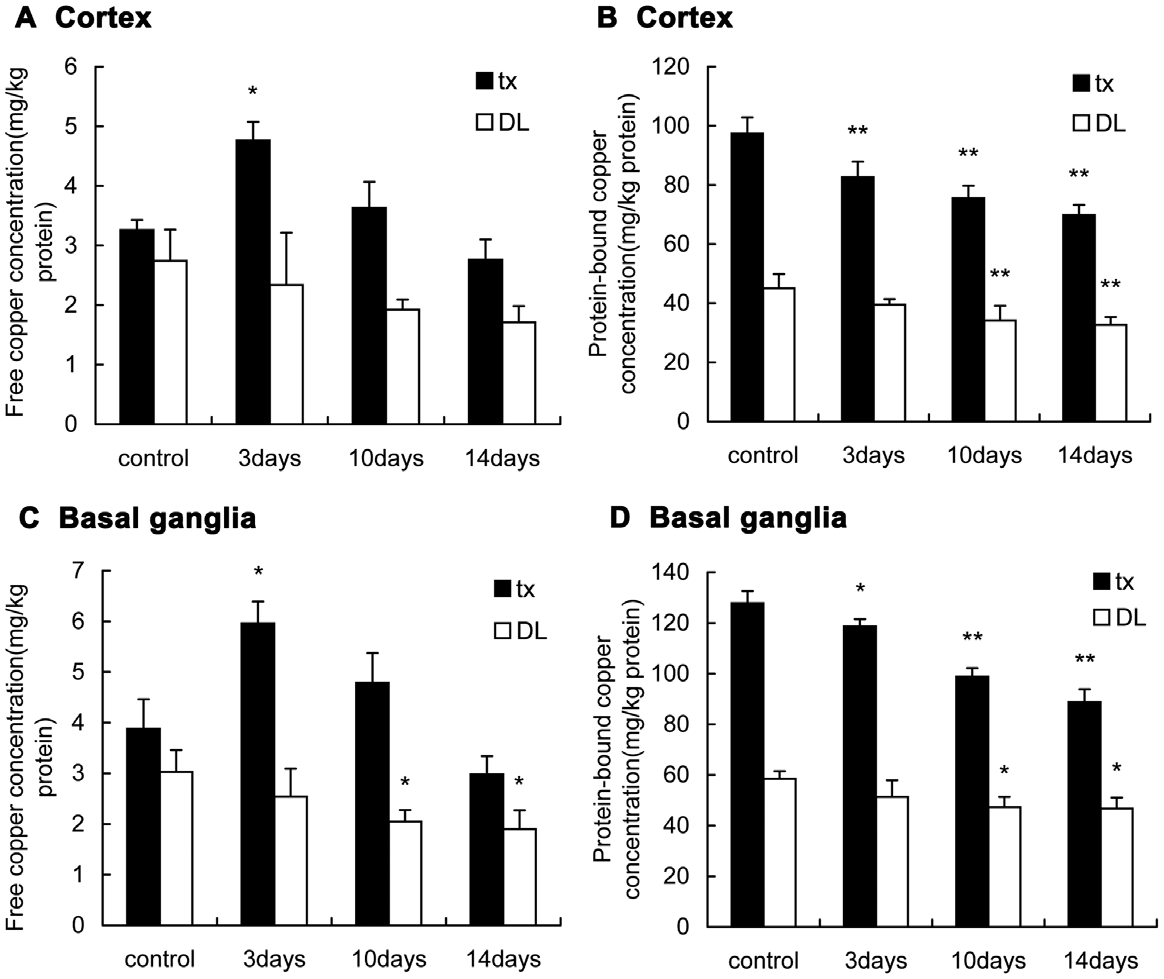
Detection of the free copper and protein-bound copper in the brain of tx and DL mice. (A)(C)The free copper concentrations in the cortex and basal ganglia of tx mice increased on the 3rd day and decreased on the 10th and 14th day,respectively. In DL mice the free copper decreased slightly along the administration. (B)(D) The protein-bound copper concentrations in the cortex and basal ganglia of tx and DL mice all decreased along the administration. The protein-bound copper concentrations in tx mice were about 2-fold higher than that of DL mice. (* P<0.05; ** P<0.01).

Figure 2 shows the free copper concentrations in the cortex and basal ganglia of tx mice were slightly higher than that of DL mice and also it shows the same tendency as that of the serum after administration, which significantly elevated (P<0.05)on the 3rd day and gradually lowered on the 10th and the 14th day. The free copper concentrations in DL mice were slightly decreased during the treatment. The protein-bound copper concentrations in the cortex and basal ganglia of the control tx mice were approximately 2-fold higher than that of DL mice,and gradually decreased along the administration compared with the control group (P<0.01). On the 14th day the protein-bound copper concentrations almost 25% decreased, while in DL mice the concentrations also slightly decreased during the administration.

### Relative mRNA expression of ATP7A and CTR1

**Figure 3:**
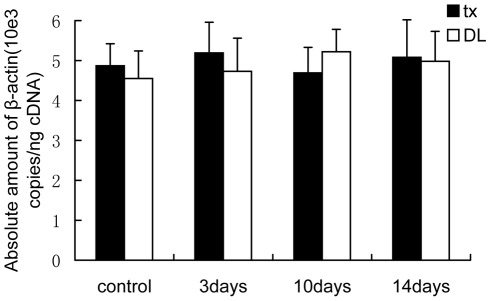
Absolute quantitation of β-actin in the cDNA from tissue. The copies of β-actin analyzed by absolute standard curve method did not change prominently in the basal ganglia of tx mice or DL mice during PA treatment.

To make sure β-actin mRNA expression was unchanged during the PA treatment,the expression stability was analyzed by absolute standard curve method. The copies of β-actin did not change prominently in the cortex and basal ganglia of tx mice or DL mice during the PA treatment. Figure 3 showed the stable expression of β-actin mRNA in the basal ganglia of the mice.

**Figure 4:**
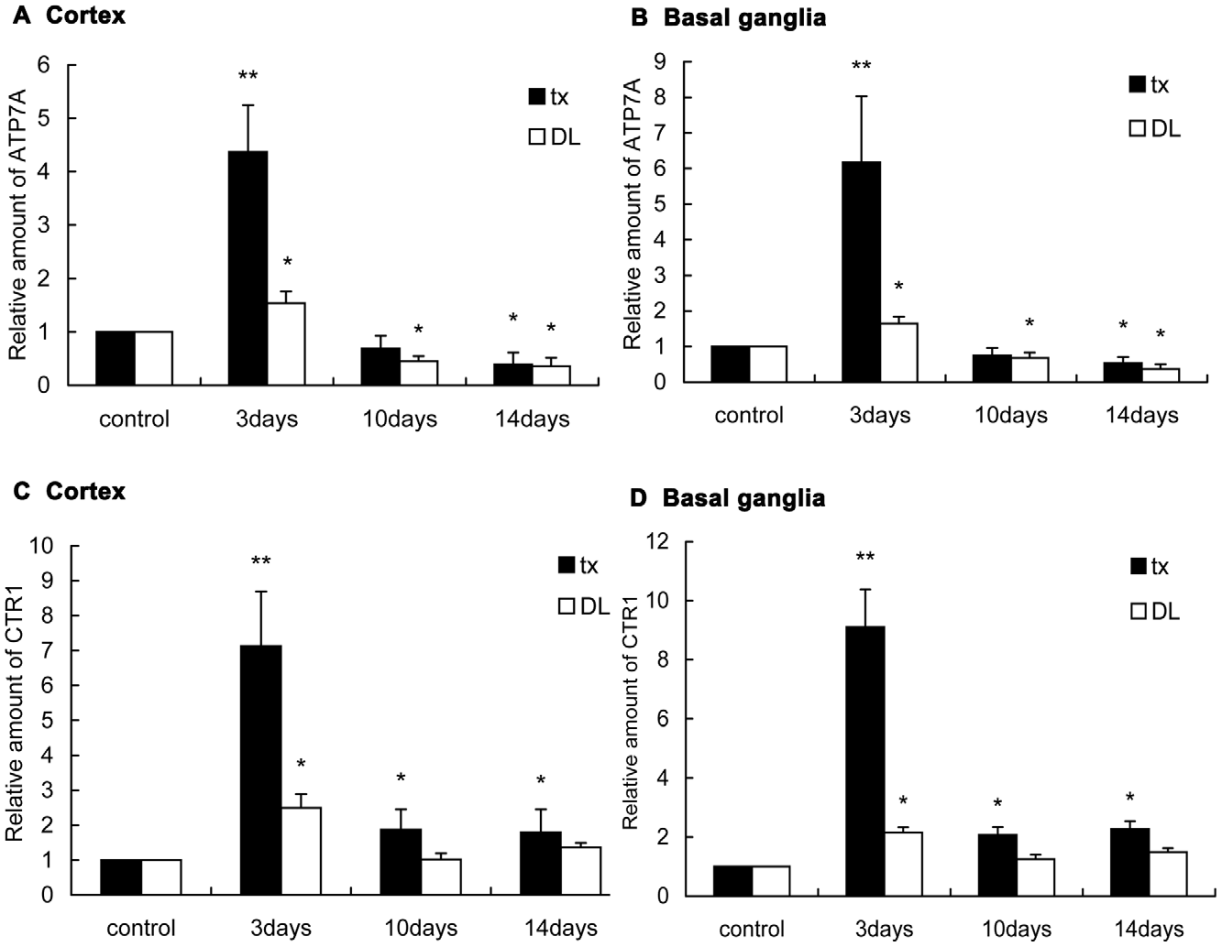
ATP7A and CTR1 mRNA expression in the brain of tx and DL mice. (A)(B)(C)(D)The ATP7A and CTR1 mRNA expression in the cortex and the basal ganglia increased significantly on the 3rd day, and declined close to the level of the control on the 10th and the 14th day. In DL mice mRNA expression of ATP7A and CTR1 also transiently increased on the 3rd day. In tx and DL controls, mRNA expression value was defined as 1.0. (* P<0.05, ** P<0.01).

Relative abundance values for ATP7A and CTR1 were compared between the mice with PA administration and the controls (Fig. 4). Compared to the controls, the mRNA expression of ATP7A 4-fold increased (P<0.01) on the 3rd day in tx mice cortex after PA treatment, and 6-fold (P<0.01) increased in the basal ganglia. But on the 10th day it decreased to the level before administration, and further, lowered down to approximately 0.4-fold (P<0.05) in the cortex and 0.5-fold (P<0.05) in the basal ganglia as high as the controls on the 14th day. In DL mice,ATP7A mRNA expression 1.7-fold increased (P<0.05) in the cortex and 1.8-fold (P<0.05) in the basal ganglia on the 3rd day, and 0.5- and 0.7-fold decreased (P<0.05) in the cortex and basal ganglia on the 10th day, respectively, while 0.4-fold decreased (P<0.05) in both locations on the 14th day.

CTR1 mRNA expression increased by 6.9-fold (P<0.01) in the cortex and 9.1-fold in the basal ganglia of tx mice on the 3rd day after administration, but on the 10th day, it decreased significantly. Compared to controls, CTR1 expression increased by 1.9-fold (P<0.05) in the cortex and by 2.1-fold (P<0.05) in the basal ganglia on the 10th day, while by 1.8- and 2.3-fold (P<0.05) on the 14th day in the cortex and basal ganglia, respectively. In DL mice, CTR1 expression increased by 2.5-fold (P<0.05) in the cortex and by 2.15-fold (P<0.05) in the basal ganglia on the 3rd day of administration as compared to controls. Then it dropped back to the level of the control in both locations on the 3rd and the 10th day.

### Immunofluorescence of ATP7A and CTR1

**Figure 5:**
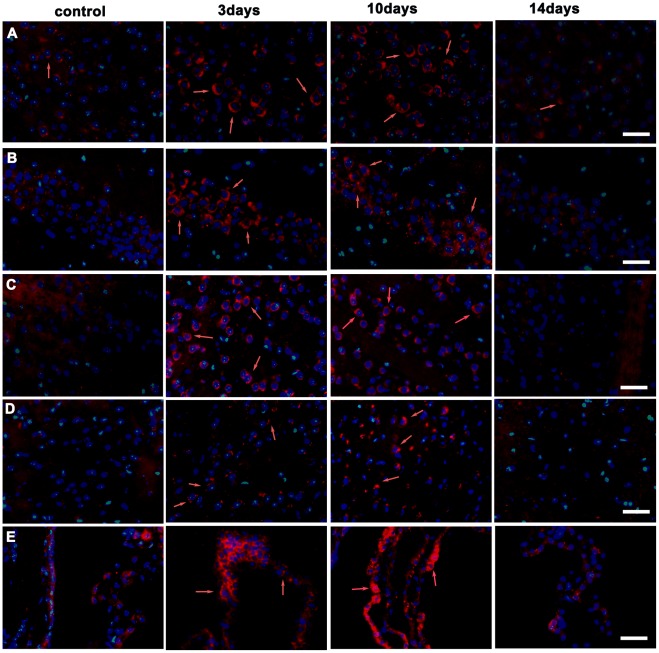
Detection of ATP7A by immunofluorescent staining (red) in the coronal brain slices of tx mice. Tx mice were subjected to PA administration for 3 days, 10days and 14days, respectively. (A) Internal pyramidal layer. ATP7A immunoreactivity staining was weak in internal pyramidal layer neurons in the control mice cortex, but increased significantly on the 3rd and the 10th day, then decreased on the 14th day. (B)hippocampus CA1 region, (C)caudate putamen(CPu), (D) central medial thalamic nucleus(CM),(E)choroid plexus in the lateral cerebral ventricles.ATP7A staining was weak in cells of hippocampus CA1 region, CPu, CM and choroid plexus in the control mice, and significantly increased on the 3rd day and the 10th day during PA administration, and declined on the 14th day. Scale bars = 30 um.

Immunofluorescence staining of frozen brain sections with antibody to ATP7A was scarcely observed in all DL mice brain sections, but weak staining in the layer 5 pyramidal neurons in the cortex, that is, internal pyramidal layer neurons (data not shown). ATP7A antibody also stained the internal pyramidal layer neurons in the control tx mice. After PA administration, intense ATP7A staining was generally present in the brain on the 3rd and the 10th day, including the cortex, hippocampus, caudate putamen (CPu), thalamus and choroid plexus in lateral cerebral ventricles (Fig. 5), and the location with the most intense staining was the internal pyramidal layer neurons. On the 14th day, ATP7A staining generally declined in the brain sections.

**Figure 6:**
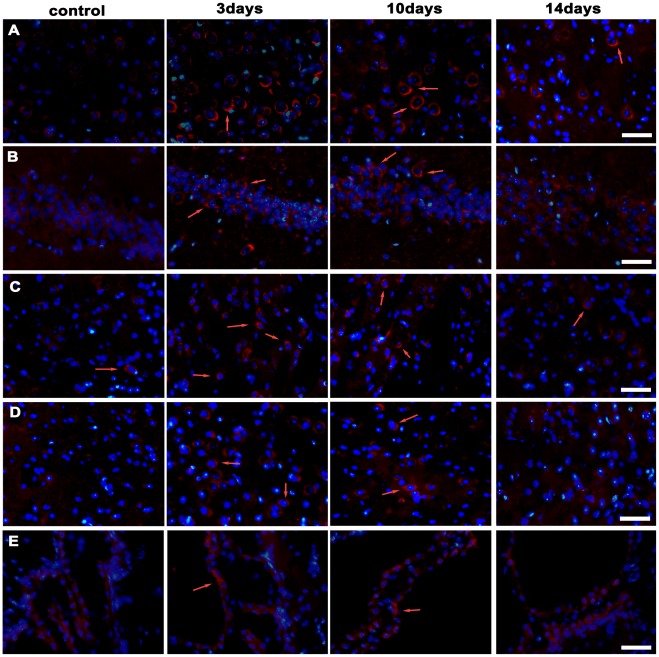
Detection of CTR1 by immunofluorescent staining (red) in the coronal brain slices of tx mice. (A) Internal pyramidal layer. There was almost no CTR1 immunoreactivity staining in the internal pyramidal layer neurons in control mice cortex. The staining presented on the 3rd and the 10th day, but declined on the 14th day during PA treatment. (B) hippocampus CA1 region, (C) caudate putamen (CPu), (D) central medial thalamic nucleus(CM). CTR1 staining was scarcely observed in cells of hippocampus CA1 region, CPu, and CM in the control mice, but presented on the 3rd and the 10th day during PA treatment, and declined on the 14th day. (E) choroid plexus in lateral cerebral ventricles. There was rare staining in the choroid plexus cells in tx mice with or without PA administration. Scale bars = 30 um.

Immunofluorescence staining of CTR1 showed the staining was seldom present in the brain tissues of DL mice with or without PA treatment (Data not shown). CTR1 staining in the brain sections of the control group of tx mice was also scarcely observed. On the 3rd day of administration, CTR1 staining was generally but not intensely detected in the tx brain tissues, including internal pyramidal layer cells in the cortex, hippocampus, Cpu and thalamus. On the 10th day, the CTR1 staining was still present in brain cells, but almost disappeared on the 14th day. CTR1 staining was not detected in the choroid plexus or the endyma cells in tx mice brain with or without PA treatment (Fig. 6).

**Figure 7:**
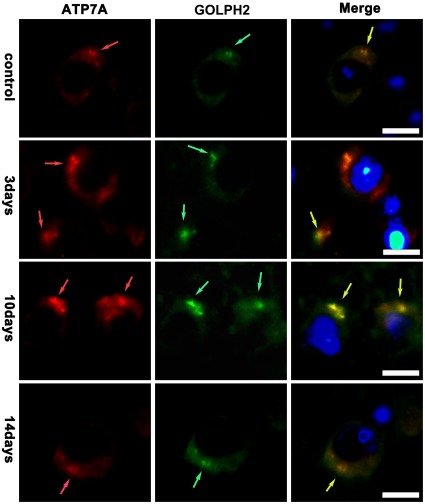
Co-localization of ATP7A and GOLPH2 in the internal pyramidal layer. The slices were from tx mice subjected to PA administration for 3 days, 10days and 14days, respectively. Golgi apparatus were labeled with GOLPH2. ATP7A(red) staining mainly co-located with GOLPH2(green) in the internal pyramidal layer of tx mice during the PA treatment, and the scope of ATP7A staining exceeded that of GOLPH2 on the 3rd day of the PA treatment. Scale bars = 10 um.

**Figure 8:**
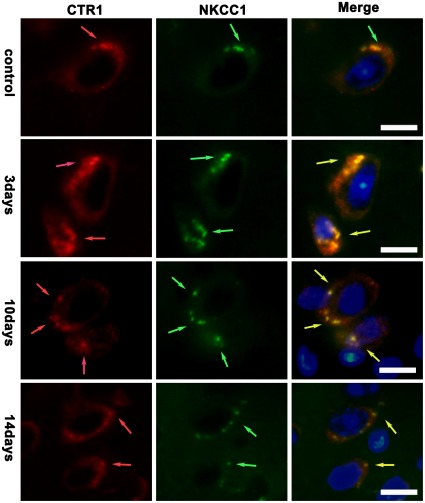
Co-localization of CTR1 and NKCC1 in the internal pyramidal layer. The slices were from tx mice subjected to PA administration for 3 days, 10days and 14days, respectively. Cellular membranes were labeled with NKCC1. CTR1(red) staining mainly co-located with NKCC1(green) in the internal pyramidal layer in cortex of tx mice during the PA treatment. Scale bars = 10 um.

Co-localization experiment showed the localization of ATP7A and CTR1 in brain cells during the PA treatment. We used GOLPH2/GOLM1, the golgi membrane protein 1, to label Golgi apparatus and viewed the co-localization of ATP7A and GOLPH2 under the oil immersion lens. Figure 7 showed that ATP7A mainly located at Golgi apparatus in the internal pyramidal layer of brain of tx mice during the PA treatment, while ATP7A staining seemed to exceed the scope of GOLPH2 staining on the 3rd day of the PA treatment. Meanwhile, we used NKCC1, the Na+/K+/2Cl− cotransporter located at cellular membrane, to label the cellular membrane. Figure 8 showed CTR1 co-located with NKCC1 in the internal pyramidal layer of cerebrum of tx mice, and it indicated that CTR1 located mainly at cellular membrane in tx mouse brain during the PA treatment.

**Figure 9:**
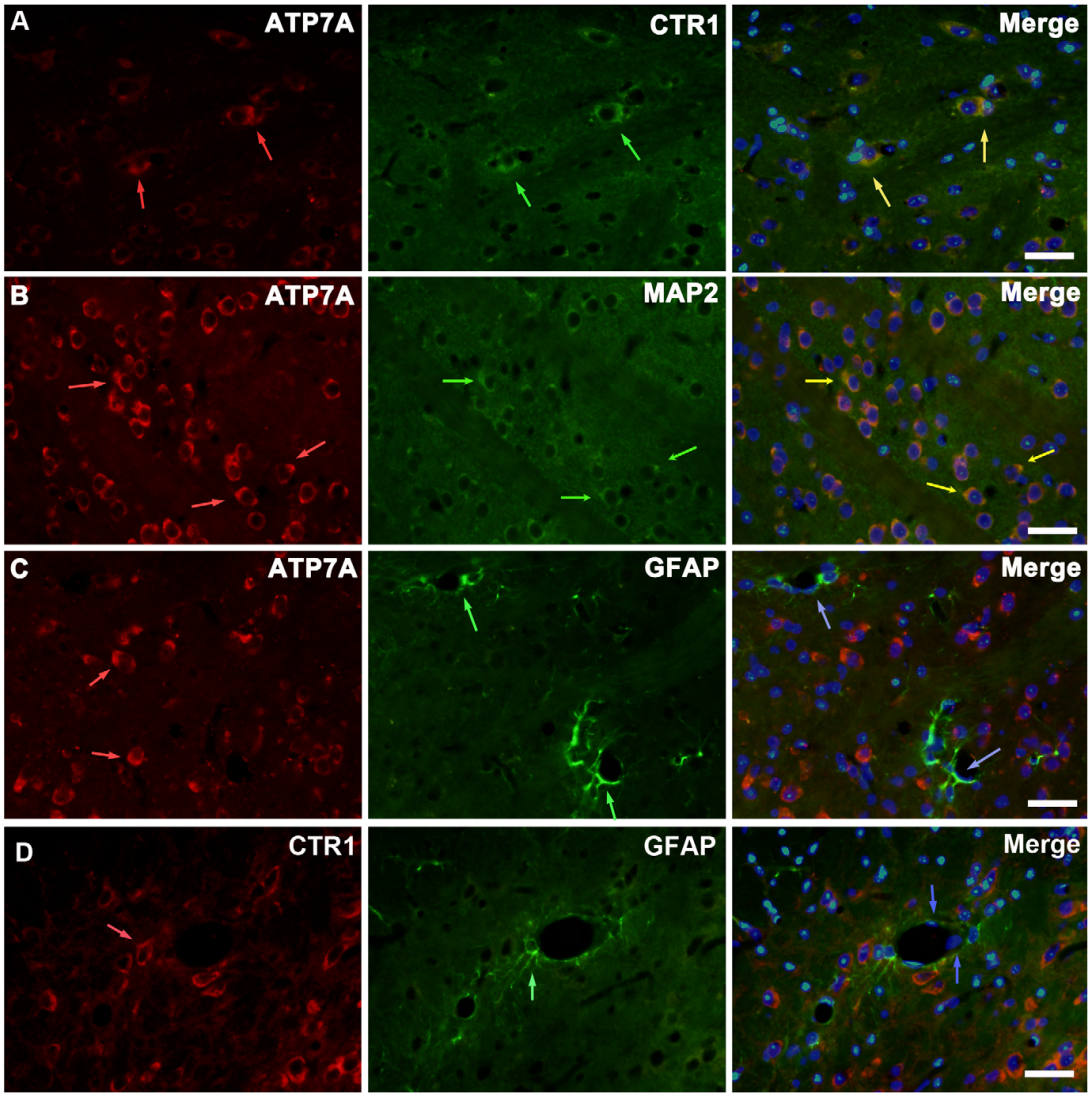
Immunofluorescent staining of ATP7A and CTR1 in neurons and astrocytes in the Cpu. The slices were from tx mice with 3 days PA administration. Neurons and astrocytes were labeled with MAP2 and GFAP, respectively. (A) ATP7A (red) immunoreactivity co-localization with CTR1 (green) in CPu (yellow arrows). (B)ATP7A (red) immunoreactivity co-localization with MAP2 (green) in CPu(yellow arrows). (C)ATP7A (red) immunoreactivity demonstrated little expression at BBB, which was formed by vascular endothelial cells (blue arrows) and astrocytic endfeet (green) ensheathing the endothelium. (D)CTR1 (red) immunoreactivity demonstrated little expression with vascular endothelial cells (blue arrows) and astrocytic endfeet (green). Scale bars = 30 um.

In brain sections of tx mice intense ATP7A staining generally located in cells expressing CTR1. Figure 9 shows the co-localization in the Cpu of tx mice with 3 days PA administration. ATP7A expression in neurons was investigated using microtubulin-associated protein 2(MAP2), which was generally detected in neurons. This suggested that PA induced increased expressions of ATP7A and CTR1 in neurons. Previous study revealed that ATP7A and CTR1 achieved to transport copper into brain through BBB. The structure basis of BBB is composed of capillary endothelium and astrocytic endfeet, which ensheath the capillary endothelium covering over 99% of the vascular endothelium. We used the cytoskeletal marker protein GFAP to identify astrocytes and astrocytic endfeet, and viewed little co-localization of ATP7A or CTR1 with GFAP as well as endothelial cells nucleus marked by DAPI in the basal lamina of BBB.

### Western blot analysis for ATP7A and CTR1

**Figure 10:**
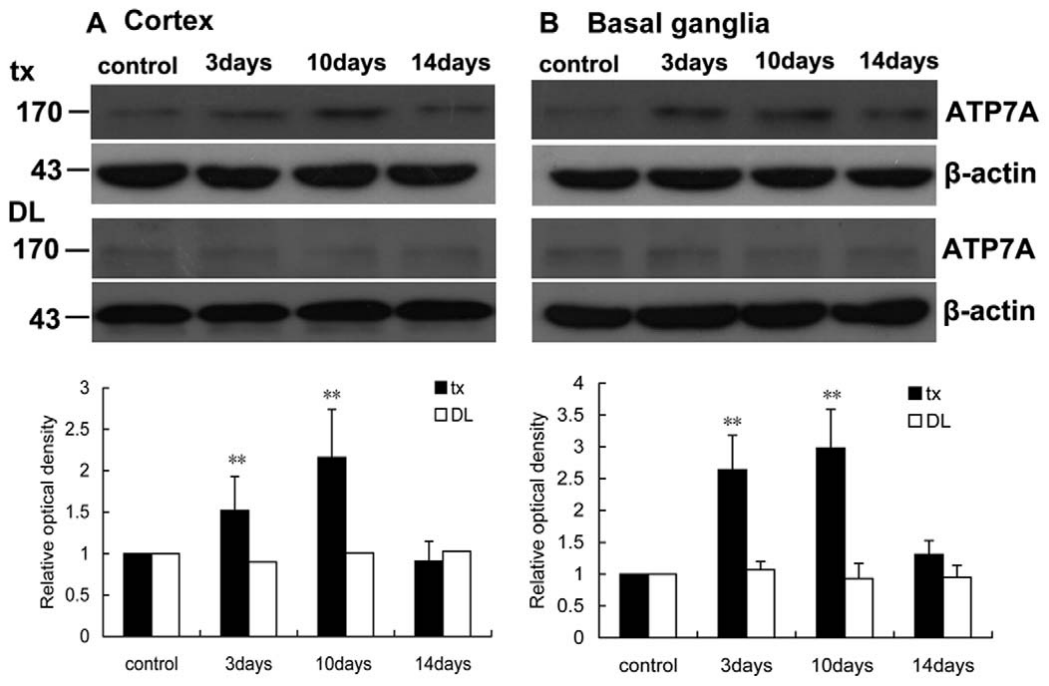
Western blot analysis of ATP7A in the cortex and basal ganglia of tx and DL mice. (A) ATP7A expression increased significantly (P<0.01) on the 3rd day and 10th day in tx mouse cortex during PA treatment, and declined closed to the level of the control on the 14th day. ATP7A expression did not change in DL mice during PA treatment. (B) ATP7A expression increased significantly (P<0.01) on the 3rd day and 10th day in tx mouse cortex, and declined closed to the level of the control on the 14th day, while it did not change in DL mice. Relative protein quantified as compared with tx or DL controls (normalized to 1.0). **p<0.01 was considered significantly different from the control.

To verify the changes of immunofluorescence staining of ATP7A and CTR1, the cortex and basal ganglia tissues of the mice with or without PA administration were examined with Western blot. The specificity of the anti-ATP7A and anti-CTR1 primary antibodies was confirmed by the protein bands with the size 170 kDa and 25 kDa, respectively. Compared with controls, the ATP7A expression 1.5-fold increased (P<0.01) in the cortex, and 2.6-fold (P<0.01) increased in the basal ganglia of tx mouse on the 3rd day after PA treatment (Figure 10). On the 10th day a 2.1-fold increased ATP7A expression was observed in the cortex as well as a more than 2.9-fold increase in basal ganglia. On the 14th day the ATP7A expression returned to the baseline in the control group. In DL mice ATP7A expression did not change in the cortex or basal ganglia during the PA administration.

**Figure 11:**
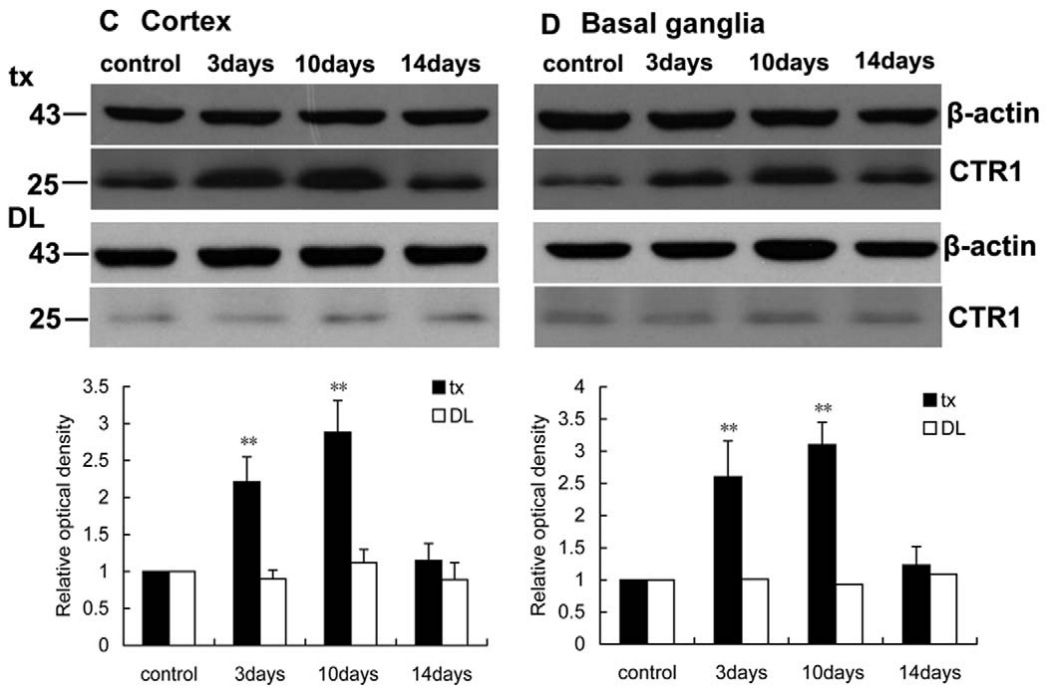
Western blot analysis of CTR1 in the cortex and basal ganglia of tx and DL mice. (A) CTR1 expression increased significantly (P<0.01) on the 3rd day and 10th day in tx mouse cortex, and declined closed to the level of the control on the 14th day. CTR1 expression did not change in DL mice during the PA treatment. (B) CTR1 expression increased significantly (P<0.01) on the 3rd day and 10th day in tx mouse cortex, and declined closed to the level of the control on the 14th day, while it did not change in DL mice. Relative protein quantified as compared with tx or DL controls (normalized to 1.0). **p<0.01 was considered significantly different from the level of the control.

CTR1 expression 2.2-fold (P<0.01) and 2.6-fold (P<0.01) increased on the 3rd day in tx mouse cortex and basal ganglia, respectively, while on the 10th day it 2.9-fold (P<0.01) and 3.1-fold (P<0.01) increased in tx mouse cortex and basal ganglia, respectively. CTR1 expression declined to the baseline on the 14th day in both regions above. In DL mice CTR1 expression did not change in the cortex and basal ganglia during the PA administration (Figure 11).

### Oxidative-stress in the central nervous system

**Figure 12:**
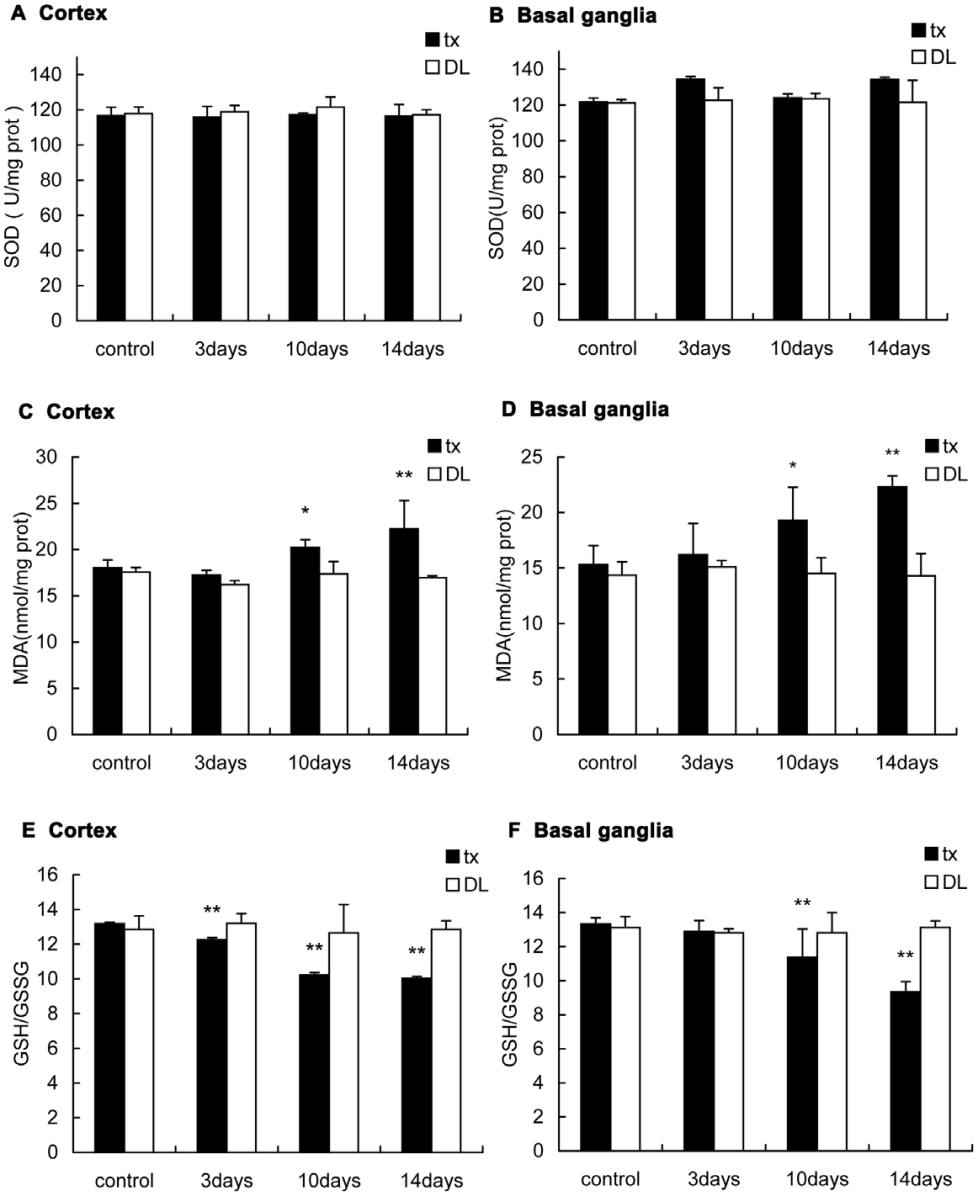
Oxidative-stress-related biomarkers assay in the cortex and the basal ganglia of the mice. (A)(B)SOD activity did not show change in tx or DL mice during the administration. (C)(D) MDA concentrations slightly increased during PA administration in the cortex and basal ganglia of tx mice but not in DL mice. (E)(F)GSH/GSSG ratios decreased gradually along the administration in the cortex and basal ganglia of tx mice, but not in DL mice. (* P<0.05; ** P<0.01).

The activity of SOD in the cortex and the basal ganglia of tx and DL mice did not change during the two weeks PA administration. MDA was measured to estimate the lipid peroxidation in the brain. The result showed that the MDA concentrations in the cortex and the basal ganglia of tx mice increased on the 10th (P<0.05) and the 14th day (P<0.01) of the administration compared to controls. But it did not show any difference in DL mice. Analysis of GSH/GSSG ratios revealed that the GSH/GSSG declined gradually in the brains of tx mice after PA administration (P<0.01). It declined from the 3rd day of administration in the cortex and from the 10th day in the basal ganglia. It did not demonstrate the variation of GSH/GSSG ratios in DL mice during PA administration (Fig. 12).

## Discussion

The neurological deterioration of WD patients during the chelator treatment has puzzled the neurologists for years. In a double blind study the spikes of the increased free copper levels in the serum associated in time with the WD patients' neurologic worsening [17]. But there are few researches on the change of copper in brain during the chelator treatment. In this study, we aimed to investigate the fluctuation of copper in the tx mice brain during PA treatment.

In our experiment the main content of serum copper in tx mice, which is the protein-bound copper, was significantly lower than DL mice. This was consistent with the reduced Cp due to the mutant ATP7B gene and verified this model. The transient increase of free copper in the serum of tx mice in the early days of PA administration was accordant with the previous report about WD patients [17]. Meanwhile, serum protein-bound copper also increased in tx mice during PA administration. This suggested that PA chelated a large amount of copper in the liver, followed by the increased copper in the serum. The mobilized copper mostly bound to the non-Cp proteins, such as albumin, which could be held-up by the ultrafiltration device and detected. When beyond the binding capability of serum proteins, the copper loosely bound to small molecules like amino acids or remained free, then filtrated through the membrane. In DL mice, the protein-bound copper was slightly decreased during the PA treatment, which might attributed to that PA reduced the copper potentially delivered to Cp by ATP7B in the liver.

In cells most of the copper is incorporated to the proteins including ceruloplasmin, cytochome oxidase, superoxide dismutase and metallothionein [18]. In our study most of the copper bound to proteins in tissue were separated by the ultrafiltration device,and the residues, which were defined as free copper, included the copper ions and the copper binding with GSH, amino acid or other small molecules. These free copper elevated at the early stage of PA administration in the cortex and basal ganglia, whereas the protein-bound copper decreased gradually. It indicated PA removed copper from the brain at the early stage of treatment and induced the increase of free copper concentrations in tissue. Herein we provided the evidence of copper mobilized by PA in the brain, though it is still not sure whether the free copper came from the brain or the liver. What is interesting is that the protein-bound copper concentrations in the basal ganglia were approximately 25 mg/kg protein higher than those in the cortex. This may be one of the reasons for the very susceptibility of the basal ganglia in WD patients.

We further documented the transient increased mRNA expression of ATP7A and CTR1 after PA administration, and the immunofluorescence results demonstrated the same tendency as transcription, while the Western blot experiment further verified the transiently increased protein expression. It has been reported a decline in ATP7A expression in the cortex from the early postnatal period to adulthood in mice, and our data verified the feature that in the adult the ATP7A staining was the most intense in layer 5 pyramidal neurons of the cortex [19]. Our data showed enhanced ATP7A expression was ubiquitously observed in the brain of tx mice 3 days after PA treatment, and still the most intense in layer 5 pyramidal neurons. The experiment revealed that ATP7A primarily expressed in internal pyramidal layer neurons, and PA induced the increase of ATP7A expression generally in the brain of tx mice but not in DL mice. It is known that all the key regulators,including ATP7A and CTR1,mediating copper homeostasis in peripheral tissues are also present in the brain [9]. When copper elevated, ATP7A left the TGN and facilitated copper excretion. Copper also induced the mRNA expression and might regulate the transcription of ATP7A in a fish cell line [20]. In our study the increased ATP7A expression was in accordance with the concentrations of free copper,which suggested that the elevated expression of ATP7A may be induced by the increased copper in the brain on the early phages of PA treatment. Co-localization experiment revealed that ATP7A mainly located at Golgi apparatus during the PA treatment, while the scope of ATP7A immunostaining exceeded that of GOLPH2 staining in brain cells of tx mice on the 3rd day of the PA treatment. It indicated the alteration in subcellular localization of ATP7A during the PA treatment,which might result from the trafficking of ATP7A from TGN to the vesicles induced by increased copper concentration.

The same tendency of the free copper concentrations in the serum and the brain tissues during PA administration brought about the question that whether the increased free copper in the brain came from the serum, or directly mobilized from the brain cells. The mechanism of copper transporting into the brain is still unclear. Previous data showed that the ATP7A and CTR1 expressed more profoundly in the brain barrier fractions (cerebral capillaries and choroid plexus) than in the brain parenchyma, and the copper transported into the brain primarily occured via the BBB as free ions [11], [12]. Our data revealed most of the copper mobilized by PA in the serum was bound to proteins. If the free copper in the brain came from the serum, the copper transporters at BBB should be observed. In fact we found CTR1 and ATP7A hardly expressed in cells around BBB, which didn't support the hypothesis that the free copper originated from the serum. Compared with the weak signal before PA treatment, the ATP7A signal was abundant in the choroid plexus during two weeks treatment. It was likely that ATP7A played a role on regulating copper concentration in the brain and the CSF. The findings of in situ brain perfusion studies suggested copper was utilized and released into the CSF via the interstitial fluid and removed by the BCB from the CSF to blood [12]. Subsequently the increased expression of ATP7A might suggest that ATP7A exported the excess copper mobilized by PA from the brain cells, then the free copper increased in interstitial fluid which in turn led to the higher expression of ATP7A in the choroid plexus and secreted more copper into CSF.

CTR1 transcription didn't appear to change as a function of Cu status in mammal cells [21], [22]. But in our work CTR1 staining and protein expression paralleled the transient increase of the mRNA expression with the increased signal in the cortex and the basal ganglia during the PA treatment, especially in layer 5 pyramidal neurons. CTR1 mostly located at the plasma membrane,where it functioned in uptake of copper. Its increased expression conflicted with the increasing of ATP7A, which played the opposite role of CTR1. This phenomenon may be explained by the following feedback mechanism. The brain cells of tx mice had been accommodated to the copper accumulation for long. However, the copper was quickly and indiscriminately chelated by PA, and the protein-bound copper, which performed the major physiologic function in the whole cells, decreased quickly during the PA treatment. Therefore, cells had to ingest the extracellular copper, reactively, to adjust the change and to meet the physiologic needs.

We suggested that the excessive oxidative stress might occur in the brain of tx mice after PA treatment. Copper induces oxidative stress by two mechanisms. First, it was suggested that reaction of Cu(I) with hydrogen peroxide and re-reduction of Cu(II) by superoxide via Fenton and Haber-Weiss chemistry yielded hydroxyl radicals [23], [24], [25]. Second, exposure to excessive copper significantly decreases the glutathione level which is the major water-soluble antioxidant in cells and directly reduces most of the ROS [26]. Copper-overloaded rats exhibited oxidative injuries with decreased GSH and increased MDA in the brain [27], [28]. MDA also increased in the livers of rats with elevated copper [27]. In our study GSH/GSSG ratios decreased in the cortex and basal ganglia and this indicated declined reductive capacity and antioxidation during PA treatment. The increased MDA, the lipid peroxidation index, implied the increased oxidative stress and cellular damage. Though MDA and GSH/GSSG decreased, the SOD levels remained in our study. SOD protected cells from oxygen free radical by catalyzing the removal of O_2_^−^. Now the change of SOD activity induced by copper was not unanimous in different studies [28], [29], [30], [31]. Different copper concentrations may result in different adjustment of the activity of SOD. Whether SOD is involved in the defence of copper-induced oxidative damaged is still controversial.

The tx mice did not demonstrate neurologic symptoms for lifetime. Nevertheless, in our study we found the evidence of increased free copper and increased oxidative biomarkers in the tx mice brain during PA administration. It suggested the neurotoxicity of PA administration in tx mice, even which didn't showed significant abnormalities of central nervous system. We presume two weeks administration may be too short to lead to prominent brain damage. Some of WD patients didn't demonstrate neurologic worsening during PA treatment, even suffered to obvious neuroligic symptoms. We suppose other factors, such as mutation styles, involved in the brain reacting to the chelator treatment.

In conclusion, the present study revealed the elevated free copper and declined protein-bound copper concentration in the cortex and basal ganglia of tx mice accompanied by general transiently increased of ATP7A and CTR1 in the brain during PA treatment. The scarce expression of ATP7A and CTR1 in the BBB and the increased ATP7A in choroid plexus suggested that the increased free copper in the brain might be mobilized from the brain parenchyma but not the serum. The declined oxidative biomarkers GSH/GSSG ratio and MDA concentrations indicated the oxidative stress raised by chelator treatment and this might be due to the increased free copper in the parenchyma. The increased free copper and oxidative stress partly explains the neurological deterioration of WD patients with PA treatment.

## Materials and Methods

### Ethic statement

All animal procedures were conducted in accordance with and approved by the animal ethics guidelines of the Institutional Animal Care and Use Committee of Sun Yat-sen University (ethics approval number20100603005). Strict attention was given to the care and use of animals according to Chinese Law. All efforts were made to minimize the number of animals used and their suffering.

### Animals

Toxic milk mutant mice (tx mice) and control mice (DL mice) were kindly presented by professor Julian. Mercer(School of Life and Environmental Sciences,Deakin University, Australia) and arose in the Laboratory Animal Center of Sun Yat-sen University, Guangzhou, China. Mice were housed in a temperature-controlled, 12∶12 light/dark room and were allowed free access to water and food. Pups of tx mice required fostering in order to survive and were routinely cross fostered within 5 days of birth to DL mice that had produced litters at the similar time. All mice used in this study were 16 weeks old (the peak time of copper deposition in tx mice).

### Reagents and antibodies

The chemical was purchased from the following sources: D-Penicillamine (PA)(sigma), antibodies for immunofluorescence: goat anti-ATP7A antibody(goat anti-human), goat anti-NKCC1 antibody(Santa, USA), rabbit anti-CTR1 antibody(Novus biologicals, USA), rabbit anti-GOLPH2 antibody(Beijing Biosynthesis Biotechnology, China), mouse anti-microtubulin-associated protein 2(MAP2) antibody (Boster, China), mouse anti glial fibrillary acidic protein (GFAP) antibody (Cell signaling technology, USA). The following were used as secondary antibodies: Cy3-conjugated goat antirabbit immunoglobulin (Proteintech group, USA); Cy3-conjugated donkey antigoat immunoglobulin (Proteintech group, USA); and FITC-conjugated goat antimouse immunoglobulin (Proteintech group, USA), antibodies for Western blot: chicken anti-ATP7A antibody (Abcam,USA), rabbit anti-CTR1 antibody(Novus biologicals, USA), secondary antibodies: peroxidase anti-chicken IgG (Proteintech, USA), peroxidase-conjugated anti-rabbit IgG.

### Experiment design

PA was dissolved in deionized water and given to mice (tx and DL mice) with intragastric administration at doses (200 mg/kg/d) in 2 divided dosages for 3 days, 10 days or 14 days respectively. Tx mice with PA were model intervention groups, and DL mice with PA were normal intervention groups (n = 5 for each time point in each index as following). Mice (tx and DL mice) given with deionized water through intragastric administration were taken as blank control groups.

### Serum and tissue free and protein-bound copper concentration analysis

Animals (n = 5 for model intervention group and normal intervention group, n = 3 for both blank control groups, in each time point) euthanized by injection of chloral hydrate (350 mg/kg body). Blood samples were taken from the retrobulbar plexus. For serum preparation blood samples were centrifuged at room temperature for 10 mins. Animals were sacrificed by decapitation and cortex and basal ganglia samples were collected. Brain samples were homogenized in ten times the volumes of the buffer (50 mM Tris buffer, PH 7.4), and centrifuged for 60 mins at 100000 g at 2°C [32]. The protein concentration of supernatant was quantitated by BCA protein assay kit (Thermo Scientific). The supernatant and serum samples were ultrafiltrated by the ultrafiltration centrifugal filter devices(Millipore, 3 kD) with low binding regenerated cellulose membrane. To retain albumin which binds much of the copper in blood except ceruloplasmin, and metallothionein which binds most of the copper in cells, we selected the type of 3 kD. The proteins larger than 3 kD molecular weight in the serum and homogenate of cortex and basal ganglia were held up by the ultrafiltration centrifugal filter devices (Millipore, 3 kD) in the concentrate. The copper being free ion and the copper binding with small molecules were in the filtrate (in this work we defined it as free copper). The filtrate and the concentrate were wet-ashed with the same volume of nitric acid. And copper concentrations were determined by air/acetylene flame atomic absorption spectrometry, using a Hitach Z5000 spectrophotometer.

### Real-Time quantitative PCR

Animals were treated with PA or deionized water as described above (n = 5 for model intervention group and normal intervention group, n = 3 for both blank control groups, in each time point). Cortex and basal ganglia tissues were homogenated in Trizol reagent and total RNA was isolated. Two micrograms of RNA was used to synthesize single-strand cDNA by PrimeScript RT-PCR Kit (Takara, Japan).The resulting cDNA was used for Real-time PCR gene expression analysis of CTR1 and ATP7A. The specific primers for Atp7A(5′-AAACCTTGCGAGAAGCAATTG, and 5′-GGGCA AAAGAGGTGTTTCCA),CTR1 (5′- TGATGATGATGCCTATGACCT, and 5′-G AAGATAGCCCGAGAGGGTC) and β-actin (5′-GACAGGATGGCAGAAGGA GATTACT, and 5′-TGATCCACATCTGCTGGAAGGT). Real time PCR was performed in triplicate on a DNA Engine Option (MJ Research) using a SYBR Green I kit (Takara, Japan).The following protocol was used for all genes: denaturation: 10 min-95°C;amplification and fluorescence acquisition (36 cycles): 15 s — 95°C, 10 s 60°C; 10 s — 72°C with a single fluorescence measurement; melting curve: from 40°C to 99°C, 0.1°C/s heating rate and continuous fluorescence measurement. Specificity of all products was verified by melting-curve analysis and by analysis on agarose gels. Abundance data for each gene is expressed as fold change. Samples were run in triplicate on each plate and the results were analyzed with the relative standard curve method. To make sure β-actin is a suitable reference gene,the expression stability was analyzed at the beginning of the experiment. A pair of specific primers for β-actin (TGCTGTCCCTGTATGCCTCT,TCATCGTACTCC TGCTTGCT) was designed. The PCR product (676 bp) purified by E.Z.N.A®. Cycle-Pure Kit (Omega, USA) was determined by Beckman-coulter DU 800 spectrophotometer for the DNA concentration. Total DNA copies were calculated by a free software offered online by URI Genomics & Sequencing Center (http://www.uri.edu/research/gsc/resources/cndna.html), and purified PCR product was used as defined template of β-actin to perform a standard curve. The copies of β-actin in the cDNA from tissues was analyzed by absolute standard curve method.

### Immunofluorescence of CTR1 and ATP7A

Animals were treated with PA or deionized water as described above (n = 5 for model intervention group and normal intervention group, n = 3 for both blank control groups, in each time point). Mice were anesthetized with chloral hydrate (350 mg/kg body) and perfused in the left ventricle with saline followed by 4% polyoxymethylene in 1×PBS, pH 7.4. The brain was extracted and post-fixed 6 h in 4% polyoxymethylene, then incubated overnight in 20% sucrose, then 30% sucrose and then embedded in Tissue-Tek Optimal Cutting Temperature compound(Sakura Fine Technical, Tokyo, Japan)at −20°C. Coronal section of 10 um were sliced and placed onto poly-L-lysine-coated slides. Slides were rehydrated in phosphate-buffered saline (PBS), and then permeabilized with 0.03% Triton X-100 in PBS for 15 min at room temperature. Slides were washed in PBS and blocked for 1 h at room temperature using 5% bovine serum albumin in PBS. Slides were then incubated overnight at 4°C with primary antibody (CTR1 at 1∶100 or ATP7A at 1∶300) in dilution buffer (1%BSA, in PBS). Unbound antibodies were removed by washing in PBS. After that the slides were incubated for 1 h in the dark with fluorescein isothiocyanate (FITC)-conjugated IgG, or Cyanine3 (Cy3) -conjugated donkey IgG. After a further wash in PBS, the slides were incubated with 4,6-diamidino-2-phenylindole (DAPI) at 500 ng/ml diluted in PBS for 15 minutes, and analyzed using an Olympus microscope.

### Western blot analysis

Mice were sacrificed (n = 4 for model intervention group, n = 3 for normal intervention group) and tissues were prepared as previously described. Proteins (50 ug) were boiled in reducing buffer (final concentrations:16.7 mmol/L Tris-HCl, pH 6.8, 2% SDS, 0.83% b-mercaptoethanol,3.3% glycerol, and 0.016% bromophenol blue), electrophoresed in(8% for ATP7A, 10% for CTR1)SDS-PAGE gel (120 V) and transferred to Polyvinylidene fluoride membrane (Roch) using 100v for 90 min. Membranes were blocked with 5% skim milk for 1 h at room temperature, and incubated with the chicken anti-ATP7A antibody (1∶1000, Abcam, USA) and the goat anti-CTR1 (1∶1000, Novus biologicals, USA), respectively, in Tris-buffered saline containing 0.1% Tween-20 (TBST) at 4°C for 2 h. Membranes were then washed with TBST and incubated for 1 h at room temperature with peroxidase-conjugated anti-goat IgG or anti-chicken IgG (1∶3000, Proteintech, USA) in TBST. Proteins were detected by chemiluminescence (Super ECL Plus Detection Reagent, Applygen, China). Densitometry was used to evaluate immunolabeled protein intensity. Pixel intensities were quantified using Multi Gauge software V2.2 (Fujifilm) and their levels were normalized against β-actin controls.

### Oxidative-stress-related biomarkers assay

#### Sub-brain homogenate preparation

Animals were treated with PA or deionized water as described above (n = 5 for model intervention group and normal intervention group, n = 3 for both blank control groups, in each time point). The cortex and basal ganglia of the mice were homogenized in 0.1 mol/L PBS(PH7.3) at 4°C. The homogenate was centrifuged with 10000×g for 10 min at 4°C and the supernatant was collected for oxidative stress biochemical assay. The supernatant was subjected to the measurement of the protein contents. We used the BCA protein assay kit (Thermo Scientific) to quantify protein concentrations. Oxidative-stress-related biomarker levels were then normalized to milligram protein.

#### Assessment of SOD levels

The activity of superoxide dismutase (SOD) was measured by a modified nitrite method with a commercial-available kit (Nanjing Jiancheng Bioengineering Insititute, Jiangsu, China). Superoxide generated by hypoxanthine and xanthine oxidase was transformed to nitrite ion by hydroxylamine. Nitrite ion was measured by color densitometry at 550 nm using a coloring reagent. The least necessary amount of SOD inhibited the rate of nitrite ion generation by 50% was defined as 1 U of SOD.

#### Assessment of MDA levels

For lipid peroxidation assay, we used a colorimetric microplate assay kit (Beyotime Institute of biotechnology, Zhejiang, China) to quantify the generation of malondialdehyde (MDA) according to the protocol. MDA was determined by measuring the red product, thiobarbituric acid(TBA)-MDA, generated by TBA and MDA at 100°C for 15 min at 532 nm absorbance.

#### Assessment of GSH/GSSG ratio

The concentrations of total glutathione (T-GSH),reduced glutathione(GSH) and oxidized disulfide(GSSG) were measured by colorimetric microplate assay from the kits(Beyotime Institute of biotechnology, Zhejiang, China).The T-GSH level was determined by the method of 5,5-Dithio-bis(2-nitrobenzoic acid)(DNTB)-GSSG recycling assay. GSSG level was measured by measuring the 5-thio-2-nitrobenzoic acid (TNB) which was measured at 412 nm absorbance. The amount of reduced GSH was determined by subtracting the amount of GSSG from the total GSH. Then the GSH/GSSG ratios were calculated.

### Statistics

Data presented in this work corresponded to the arithmetical mean±SEM. Statistical significance (one-way ANOVA, followed by post hoc Bonferroni) was performed using SPSS 13.0. Values were considered to differ significantly at the level of p<0.05.
