# Correction: Penicillamine Increases Free Copper and Enhances Oxidative Stress in the Brain of Toxic Milk Mice

**DOI:** 10.1371/annotation/e57c1988-71fd-4809-8d84-aff56e60b2c7

**Published:** 2012-08-13

**Authors:** Ding-Bang Chen, Li Feng, Xiao-Pu Lin, Wei Zhang, Fu-Rong Li, Xiu-Ling Liang, Xun-Hua Li

The following information was missing from the Funding section: This study was also supported by the National Natural Science Foundation of China grant #81171070.

